# CRISPR cascade: edited fungal polys for idiopathic pulmonary fibrosis with emphysema mimics

**DOI:** 10.1097/MS9.0000000000004362

**Published:** 2025-11-14

**Authors:** Iman Osman Abufatima, Umair Ali

**Affiliations:** a Faculty of Medicine, University of Medical Sciences and Technology, Khartoum, Sudan; b Department of Pharmacy, University of Swabi, Khyber Pakhtunkhwa, Pakistan

*To the Editor,*

Renewed interest in CRISPR-Cas-based control of fungal polysaccharide production holds new horizons in antifibrotic treatment with a specialty interest in idiopathic pulmonary fibrosis (IPF) models featuring emphysema-like remodeling^[1]^. The paradigm of CRISPR Cascade complexes as precise editors of fungal genomes offers a unique opportunity to reprogram the immunologic and structural interfaces that underpin pulmonary fibrosis^[2]^. This letter aims to highlight the emerging mechanistic rationale, translational potential, and current limitations of CRISPR-edited fungal polysaccharides as novel modulators in fibrotic-emphysematous disease.

Fungal polysaccharides such as β-glucans, mannans, and chitin are increasingly recognized as bioactive molecules that orchestrate immune-fibroblast cross-talk^[3]^. Such polymers can potentially regulate alveolar macrophage polarization, fibroblast activation, and extracellular matrix (ECM) remodeling via cytokine circuits involving TGF-β, IL-13, and IL-10^[4]^. By leveraging CRISPR-Cas systems, whether Cas3-mediated repression of multigene or Cascade complexes for transcriptional control, biosynthetic pathways can be precisely engineered to suppress pro-fibrotic glycosylation motifs, induce anti-inflammatory conformations, or make immunologically inert scaffolds^[5]^. This molecular engineering is outside of conventional antifungal or immunosuppressive paradigms, acting as a model for immunomodulatory biomolecules.

In IPF and emphysema, shared injury-repair processes underlie discordant ECM kinetics. Fibrosis is characterized by exuberant collagen deposition, whereas emphysema is typified by matrix destruction^[6]^. Edited fungal polysaccharides could theoretically redress this imbalance by lessening epithelial oxidative stress, inhibiting macrophage-driven inflammation, and augmenting epithelial regeneration^[2]^. Preliminary studies of precision-edited microbial glycans in organoid and murine models of fibrosis suggest that they have the ability to modulate myofibroblast differentiation and enhance resolution pathways, meriting study in combined fibrotic-destructive models^[7]^.

Translationally, CRISPR-edited fungal products can serve as adjunct antifibrotic therapies or as delivery platforms for pro-resolving mediators and stem-cell-derived vesicles^[1]^. Moreover, the platforms can also serve as in vitro models for deconstructing immune-epithelial interactions under fibrotic stress. Bioengineering platform integration can also facilitate inhalable nanocarriers from fungal biopolymers for targeted lung delivery^[4]^. Nonetheless, this technology demands rigorous biosafety evaluation, especially for fungal gene edits that may alter virulence determinants or environmental persistence.

Despite their promise, vigilance is warranted. Gene editing in fungal systems renders ethical and ecological considerations, namely horizontal gene transfers and immunogenic unpredictability. Engineered polysaccharides could influence host–microbiome dynamics or biofilm formation and will necessitate controlled *in vivo* and *ex vivo* validation before clinical translation. Multidisciplinary stewardship from molecular biologists to pulmonologists to bioethicists will be crucial in ensuring safety and reproducibility.

In conclusion, the interface of CRISPR Cascade engineering and pulmonary fibrosis biology is an innovative frontier for antifibrotic discovery. Edited fungal polysaccharides can yield immunomodulatory scaffolds that have the potential to reshape the therapeutic landscape of IPF and emphysema overlap syndromes. Further integrative research is warranted to translate this promising concept into a tangible clinical benefit.

Titan guidelines: This manuscript complies with TITAN Guidelines, 2025, declaring no use of AI^[8]^.

## Data Availability

Data sharing is not applicable to this article.

## Research registration unique identifying number (UIN)

Our study does not involve any human participants or human data; therefore, registration in a study registry is not applicable.
